# DrugGen: a database of *de novo*-generated molecular binders for specified target proteins

**DOI:** 10.1093/database/baad090

**Published:** 2023-12-27

**Authors:** Hao Qian, Jingyuan Zhou, Shikui Tu, Lei Xu

**Affiliations:** Department of Computer Science and Engineering, Shanghai Jiao Tong University, No. 800 Dong Chuan Road, Shanghai 200240, China; Department of Computer Science and Engineering, Shanghai Jiao Tong University, No. 800 Dong Chuan Road, Shanghai 200240, China; Department of Computer Science and Engineering, Shanghai Jiao Tong University, No. 800 Dong Chuan Road, Shanghai 200240, China; Department of Computer Science and Engineering, Shanghai Jiao Tong University, No. 800 Dong Chuan Road, Shanghai 200240, China; Guangdong Institute of Intelligence Science and Technology, Building 6, No. 398 Houpu Road, Zhuhai, Guangdong 519031, China

## Abstract

*De novo* molecular generation is a promising approach to drug discovery, building novel molecules from the scratch that can bind the target proteins specifically. With the increasing availability of machine learning algorithms and computational power, artificial intelligence (AI) has emerged as a valuable tool for this purpose. Here, we have developed a database of 3D ligands that collects six AI models for *de novo* molecular generation based on target proteins, including 20 disease-associated targets. Our database currently includes 1767 protein targets and up to 164 107 *de novo*-designed molecules. The primary goal is to provide an easily accessible and user-friendly molecular database for professionals in the fields of bioinformatics, pharmacology and related areas, enabling them to efficiently screen for potential lead compounds with biological activity. Additionally, our database provides a comprehensive resource for computational scientists to explore and compare different AI models in terms of their performance in generating novel molecules with desirable properties. All the resources and services are publicly accessible at https://cmach.sjtu.edu.cn/drug/.

**Database URL**: https://cmach.sjtu.edu.cn/drug/.

## Introduction

Traditional drug discovery is a laborious, expensive and time-consuming process, primarily due to the vast combinatorial complexity of the discrete molecular search space. The existing chemical space contains around 1060 drug compounds, which are more than the total number of atoms in the solar system (1). On average, it takes at least 10 years and costs $2.8 billion to develop a new drug, while only <10% of lead compounds become accessible through clinical trials and regulatory approval (2, 3).

In recent years, the advancement of deep learning methods has brought new opportunities to traditional drug design. Researchers have utilized deep generative models for *de novo* molecular design. These models are designed to learn the joint distribution between protein targets and known ligands, enabling them to efficiently generate novel molecules that can bind to specific targets.

When utilizing these models to generate molecules, users are usually required to invoke the trained models via the command line interface. On occasions where such trained models are absent, they must train these models from scratch before the generation process. This poses challenges for users from biochemistry and medical domains as they typically have limited background in machine learning and computer science. Additionally, the existing molecular generative models were evaluated on different test sets. Therefore, it is of great significance to conduct a fair and systematic analysis on the performance of these methods under a unified benchmark setting. Such analysis provides a practical guide for users on choosing an appropriate model for their task, as well as insights into the model generation process.

To address these issues, we have constructed a database, called DrugGen, of 3D ligands designed for specific protein targets and selected six prevalent models for *de novo* drug design. We have also developed a user-friendly website (https://cmach.sjtu.edu.cn/drug/) that enables users to easily access the database. By simply providing the Protein Data Bank Identifier (PDBID) and selecting the desired model, users can retrieve the corresponding ligand molecule information and download molecules either individually or in bulk. This website eliminates the need for expertise in deep learning and saves time on running models, thereby simplifying the process of generating molecules. Through these resources, we aim to facilitate the adoption of deep generative models in molecular generation and enhance the development of *de novo* drug design.

To the best of our knowledge, DrugGen is the first open-source *de novo*–designed 3D drug database entirely composed of artificial intelligence (AI)–generated models.

## Results

### Database

As mentioned in the Abstract, our current database includes 1767 protein targets and 164 107 molecules. In the future, we aim to expand our database by incorporating more experimental Protein Data Bank (PDB) structures from PDBBind (4). Completed evaluations can be found on the DrugGen website. To ensure fair evaluation of each deep generative model, we have constructed a comprehensive test set, which serves as the basis for all subsequent evaluations.

### Test set

#### Target proteins

We have utilized the data preparation and selection strategy outlined in previous studies (5, 6). This process involves disregarding poses with root mean square deviation (RMSD) of tied localization poses >1 Å, and extracting 100 proteins with <30% sequence identity as target proteins from CrossDocked (7), which is a protein–ligand database tailored for deep learning methods. In addition, as shown in Table 4, we have incorporated 20 widely recognized protein targets that are linked to various diseases, including targets for painkillers, antibiotics and those associated with coronavirus disease (COVID-19). Thus, we have constructed a test set containing 120 proteins in total to evaluate deep generative models.

#### Ligands

We have generated 61 213 ligands for proteins in test sets, utilizing six generative models, namely AlphaDrug (8), SBDD (5), Pocket2Mol (6), GraphBP (9), DiffSBDD (10) and TargetDiff (11). As shown in Table 1, we have included a comparable number of molecules for each method. Additionally, we assessed their performance using the following indicators:

**Table 1.** Numbers and properties of molecules generated by each deep learning model in the test sets

Methods	Number of molecules	Vina score	QED	SA	Log*P*
AlphaDrug	9617	**−9.77**	0.42	**0.80**	5.65
SBDD	11 339	−5.62	0.52	0.63	0.57
Pocket2Mol	13 647	−4.53	**0.58**	0.78	1.40
GraphBP	10 383	134.12^b^	0.46	0.48	3.00
TargetDiff	7118	−5.26	0.50	0.58	1.63
DiffSBDD^a^	9082	−5.26	0.45	0.30	−0.10

The property values presented in the table are the medians. Best performances are highlighted with bold values.

a Denotes that the author does not provide a trained model, and thus all results are obtained from the model that we trained by ourselves.

b Denotes that during molecule generation, GraphBP fixes the binding site range to 15 Å around the known ligand, which may cause the generated molecules to deviate from the center of the binding pocket, resulting in a bad Vina score.

- Vina score. The Vina score refers to the scoring function used in AutoDock Vina (12), a popular molecular docking software used to predict the binding poses and affinities of small molecules with its protein targets. The Vina score is a numerical value that reflects the predicted binding energy between the ligand and the receptor, with lower scores indicating stronger binding energy. The Vina score is a crucial metric in drug discovery and can serve as a key metric for virtual screening. In terms of the Vina score metric, AlphaDrug stands out from the rest and exhibits a significant improvement compared to other methods. The remaining methods, except for GraphBP, exhibit similar performance.
- Quantitative estimate of drug-likeness (QED) (13). The QED is used to evaluate the drug-likeness of a small-molecule compound, taking into account factors such as molecular weight, lipophilicity and polar surface area. The QED score ranges from 0 to 1, with higher scores indicating better drug-likeness. The QED score is used in drug discovery to filter out compounds with poor pharmacological properties. In terms of the drug-likeness metric, Pocket2Mol exhibits the best performance.
- Synthetic accessibility (SA) (14). The SA score is a metric used to indicate the level of difficulty in synthesizing a molecule, with values ranging from 0 to 1. A higher SA score indicates that the compound is easier to synthesize, whereas a lower SA score suggests that the synthesis may be more challenging. In terms of the SA metric, AlphaDrug exhibits the best performance due to its chemically reasonable structures and appropriate ring sizes.
- Octanol-water partition coefficient (LogP) (15). The LogP measures a compound’s ability to transfer between aqueous and organic phases. An ideal drug molecule typically exhibits a LogP value within the range of −0.4 to 5.6.

Furthermore, we analyzed the distribution of various ring sizes of molecules generated by each method. As demonstrated in Table 2, AlphaDrug, Pocket2Mol and TargetDiff can generate molecules with a similar distribution of various ring sizes as the reference ligands. Here, the reference ligands denote the original ligands from the protein–ligand complexes. This suggests that these methods can generate molecules with the rational size of rings. On the other hand, other methods tended to generate molecules with an over-representation of three-membered and four-membered rings.

**Table 2.** Percentage of various ring sizes for reference and generated molecules

Ring size	3	4	5	6	7	>7
Ref. (%)	1.5	0.4	28.9	66.7	0.7	1.8
AlphaDrug (%)	1.	0.4	10.9	82.1	3.3	1.4
SBDD (%)	29.5	0.1	18.0	48.5	1.7	2.2
Pocket2Mol (%)	0.1	0	17.1	78.0	3.7	1.1
GraphBP (%)	51.5	17.8	9.1	8.5	5.7	7.4
TargetDiff (%)	0	2.7	29.2	49.6	12.0	6.6
DiffSBDD (%)	70.8	5.0	9.6	7.8	3.3	3.4

To evaluate the bond length of generated molecules, we selected eight common types of chemical bonds and calculated the Jensen–Shannon divergence (16) between the bond length distribution of the generated molecules and that of the reference ligands as follows:


$$JSD(P\,||\,Q)\, = \,\frac{1}{2}{D_{KL}}(P\,||\,M)\, + {\mathrm{\,}}\frac{1}{2}{D_{KL}}(Q\,||\,M),$$

where ${D_{KL}}(P\,||\,Q)$ is the Kullback–Leibler divergence (17) between probability distributions $P$ and $Q$, and $M\, = \,\frac{1}{2}\left( {P\, + \,Q} \right)$ is the average of the two distributions. As shown in Table 3, TargetDiff achieves state-of-the-art performance on five of eight bond types, which demonstrates that the molecules generated by TargetDiff tend to have a reasonable bond length.

**Table 3.** The Jensen–Shannon divergence (JSD) between the bond length distributions of the reference ligands and generated molecules

JSD	C–C	C=C	C@C	C–N	C=N	C@N	C–O	C=O
AlphaDrug	0.43	0.83	0.46	0.64	0.83	**0.38**	0.59	**0.60**
SBDD	0.65	0.63	0.46	0.56	0.80	0.72	0.68	
Pocket2Mol	0.59	0.60	0.51	0.68	0.75	0.63	0.63	0.68
GraphBP	**0.37**	**0.53**	0.76	0.53	0.83		0.50	0.64
TargetDiff	0.48	**0.53**	**0.26**	**0.45**	0.74	**0.38**	**0.48**	0.61
DiffSBDD	0.57	0.69	0.81	0.57	**0.73**		0.59	0.70

Single, double and aromatic bonds are represented as ‘–’, ‘=’ and ‘@’, respectively. A lower value indicates better performance. Best performances are highlighted with bold values.

**Figure 1.**
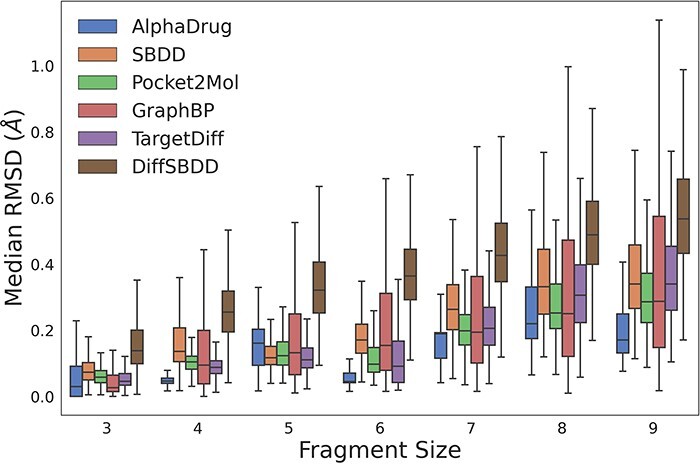
The RMSD values between coordinates of rigid fragments before and after force field optimization. A smaller RMSD value denotes a more reasonable sub-structure.

We further evaluate whether all the methods can generate molecules with reasonable sub-structures (e.g. all carbon atoms within a benzene ring lie on the same plane.). Following TargetDiff (11), we break down molecules into several rigid fragments and optimize them with Merck molecular force field (18). As shown in Figure 1, AlphaDrug tends to generate more reasonable sub-structures, followed by TargetDiff and Pocket2Mol.

Alt text: The image is a boxplot graph representing the median RMSD across different fragment sizes for various drug discovery methods. Each boxplot corresponds to a specific method, color-coded for identification, with fragment sizes on the *x*-axis and median RMSD values on the *y*-axis.

Overall, we have evaluated the molecules based on Vina score, chemical properties, distribution of various ring sizes and bond lengths of eight types. We also investigate whether generative models can produce reasonable sub-structures. We believe that this comprehensive evaluation enables users to make informed decisions based on their specific needs and requirements.

#### Case study

All aforementioned evaluations have been analyzed from a statistical perspective. In this section, we will provide a more specific evaluation of each method from a biochemical perspective, utilizing 20 concrete disease-related protein targets. By doing so, we aim to provide a deeper understanding of the performance of each method and its potential applications in the field of biochemistry and drug discovery.

Table 4 provides a comprehensive overview of 20 disease protein targets, including the number of molecules generated by each method and the median Vina scores. The table is categorized into four parts: the first 10 rows focusing on proteins related to severe acute respiratory syndrome coronavirus 2 (SARS-CoV-2), followed by 6 rows of non-steroidal anti-inflammatory drugs, 3 rows of aniline antipyretic analgesics (paracetamol) and the final row dedicated to antibiotics (penicillin). The majority of the deep learning methods produced valid molecules that were successfully evaluated by the Vina program, with TargetDiff and GraphBP achieving a 100% success rate. Besides, AlphaDrug achieves state-of-the-art performance on Vina scores, which indicates that AlphaDrug tends to generate molecules with high binding affinities.

**Table 4.** Detailed information about 20 disease-related protein targets

			AlphaDrug		SBDD		Pocket2Mol		GraphBP		DiffSBDD		TargetDiff	
Protein target	PDB id	Known ligand	Number	Vina score	Number	Vina score	Number	Vina score	Number	Vina score	Number	Vina score	Number	Vina score
3CL protease	7LKX	Y51, Y71					140	−4.1	92	139.6	84	−2.5	76	−5.3
3CL protease	7MBI	YWJ			77	−4.2	123	−4.7	86	135.6			91	−3.1
3CL protease	7R7H	4IT			113	−4.9	103	−3.9	85	128.4	92	−2.5	82	−3.4
3CL protease	7UJG	K36	78	−9.4	79	−5.8			88	132.9	93	−2.5	89	−3.2
3CL protease	8ACD	LQ6	98	−9.1	107	−4.5	103	−3.7	92	138.6	76	−2.3	82	−2.8
3CL protease	7BZ5	NAG			69	223.8	132	−1.9	84	160.4	73	−1.7	92	−2.6
3CL protease	7K9I	NAG	68	−8.3	109	80.2	141	−1.5	87	151.3	84	−2.1	87	−2.8
3CL protease	7PR0	NAG			97	83.9			86	134.9	57	−0.8	86	−3.4
3CL protease	7VNB	NAG	94	−9.2	89	131.8	116	91.6	85	156.0			88	−2.8
3CL protease	8DMA	NAG	94	−7.6	122	−1.2	108	−2.2	85	148.8	87	−0.7	82	−2.6
Phospholipase A2	1TGM	Aspirin	99	−10.0	97	141.0	109	−2.5	82	177.0			93	−3.7
Glycoprotein, SPB-40	4NSB	Aspirin	98	−9.9	68	81.9			79	79.3			94	−4.1
Myotoxin	6MQF	Aspirin	95	−3.1	87	−4.8	131	−4.4	81	119.1			76	−4.1
FABP4	3P6H	Ibuprofen	84	−9.3	116	−4.9	147	−3.5	88	147.7			91	−3.7
AKR1C2	4JTR	Ibuprofen	97	−10.9	101	−7.1	110	−4.1	89	119.5			93	−4.7
AKR1C3	3R58	Naproxen	96	−11.6	108	−6.8	132	−5.2	89	115.6			90	−4.4
Phospholipase A2	2DPZ	Paracetamol	86	−9.8	88	−2.8	105	−1.8	87	135.7	87	−3.7	91	−4.0
Human BRD2	4A9J	Paracetamol	90	−9.8	99	−3.8	141	−3.5	83	139.5			83	−3.6
Bacterial aryl acylamidase	4YJI	Paracetamol	97	−10.6	107	−3.5	103	−3.6	86	154.0	87	−3.7	87	−5.3
Penicillin acylase mutant	1FXV	Penicillin	90	−10.9	90	−6.0	119	−3.3	79	157.4			85	−4.3

Ligand notes: (i) Y71: (1S,2S)-2-((S)-2-(((((1S,2S,4S)-bicyclo[2.2.1]hept-5-en-2-yl)methoxy)carbonyl)amino)-4-methylpentanamido)-1-hydroxy-3-((S)-2-oxopyrrolidin-3-yl)propane-1-sulfonic acid. (ii) Y51: (1R,2S)-2-((S)-2-(((((1S,2S,4S)-bicyclo[2.2.1]hept-5-en-2-yl)methoxy)carbonyl)amino)-4-methylpentanamido)-1-hydroxy-3-((S)-2-oxopyrrolidin-3-yl)propane-1-sulfonic acid. (iii) YWJ: 4-methoxy-*N*-[(2S)-4-methyl-1-oxo-1-((2S)-3-oxo-1-[(3S)-2-oxopiperidin-3-yl]butan-2-ylamino)pentan-2-yl]-1H-indole-2-carboxamide. (iv) 4IT: *N*-[(2S)-1-((1E,2S)-1-imino-3-[(3S)-2-oxopyrrolidin-3-yl]propan-2-ylamino)-4-methyl-1-oxopentan-2-yl]-4-methoxy-1H-indole-2-carboxamide. (v) K36: (1S,2S)-2-(*N*-[(benzyloxy)carbonyl]-L-leucylamino)-1-hydroxy-3-[(3S)-2-oxopyrrolidin-3-yl]propane-1-sulfonic acid. (vi) LQ6: (2S)-4-[[2,4-bis(oxidanylidene)-1H-pyrimidin-6-yl]carbonyl]-1-(3,4-dichlorophenyl)-*N*-(thiophen-2-ylmethyl)piperazine-2-carboxamide. (vii) NAG: 2-acetamido-2-deoxy-beta-D-glucopyranose.

**Figure 2.**
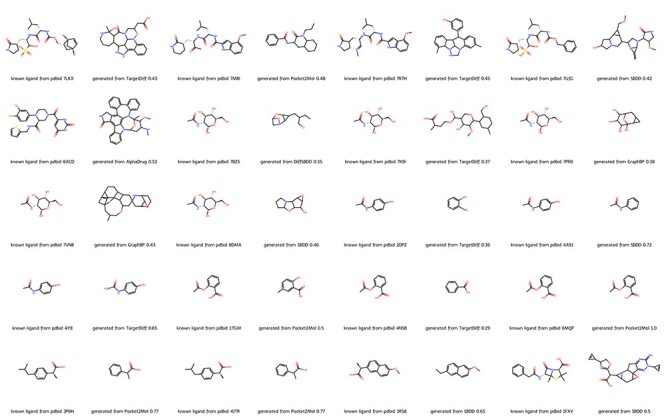
Molecules from DrugGen database with the highest similarity to the original ligands on 20 disease-related protein targets. The even-numbered columns showcase the original ligand molecules, while the odd-numbered columns display the generated molecules with the highest similarity. The values in the odd-numbered columns indicate the Tanimoto similarity to the original ligand.

We further evaluate the molecules generated by different methods from the aspect of pharmacophoric pattern (19, 20). We computed the similarity between molecules in our database and the original ligands of all 20 proteins. As shown in Figure 2, deep learning methods can generate molecules with high similarity to original ligands with small molecular weight. For example, in the case of PDBID 4YJI, the only difference between the original ligand and the molecule generated by TargetDiff is the replacement of a methyl group with an amino group. Note that Pocket2Mol successfully generated the original ligand aspirin for PDBID 6MQF.

Alt text: The image depicts a collection of molecular structures, showcasing a comparison between known ligands from PDB entries and those generated by computational models such as TargetDiff, AlphaDrug, Pocket2Mol, GraphBP, DiffSBDD and SBDD. Each row presents a pair of molecules, with the known ligand on the left and the generated ligand on the right, along with the associated PDB codes and model performance metrics, i.e. the Tanimoto similarity.

#### DrugGen website

**Figure 3.**
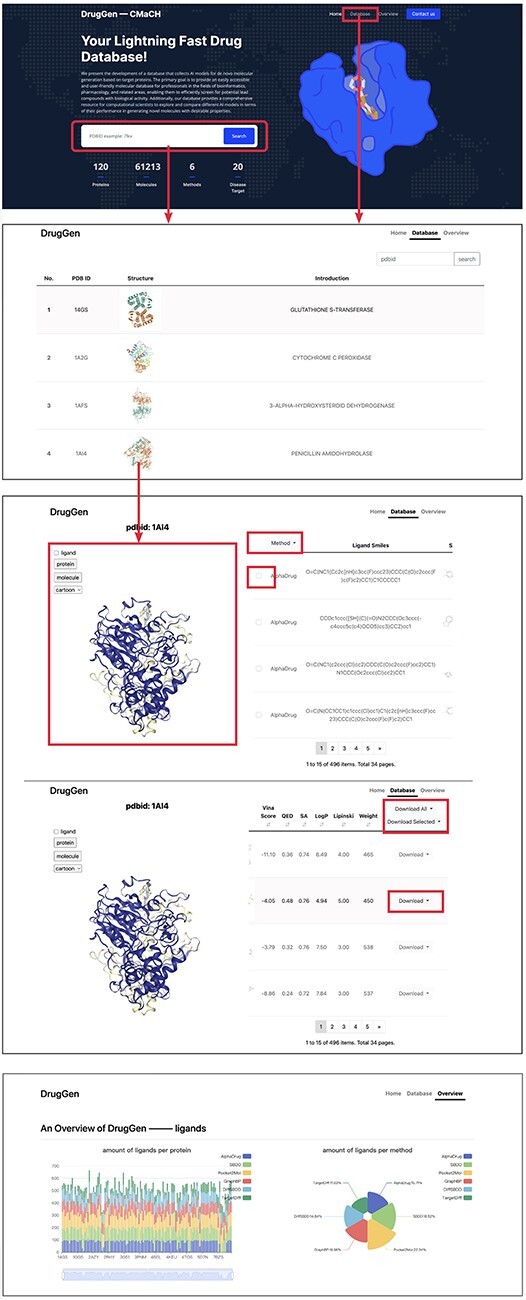
Quick tutorials about utilizing the website to obtain molecules on specific protein targets. We highlighted main interfaces with rectangular boxes. The bottom image displays part of the ‘overview’ page, which delivers a comprehensive evaluation of each method.

In this section, we provide a brief guide on how to use our website. As illustrated in Figure 3, the first image with a deep blue background displays the homepage of our website, while the second image shows the protein list page, and the third image aims to provide more detailed information across each protein.

Alt text: The image shows a guide for navigating the database, detailing the steps to search for and visualize the binding pose between a molecule and a target protein.

#### Home page

On the homepage, we have highlighted main interfaces with red boxes. The first interface is the search box which enables users to input a PDBID to navigate to the protein details page or click on ‘Database’ on the navigator bar to access the protein list page. Below the search box, a brief overview of our database and the quantity of available data are provided.

#### Dataset page

The dataset page lists all the proteins in the database, including their PDBIDs, 3D structures and general descriptions. Users can search proteins using the search box located at the top right corner. Clicking on a row, such as the row where ‘1AI4’ is located, leads to the third page, where the left viewport displays the 3D structure of the protein ‘1AI4’, and the right table lists the simplified molecular input line entry specification and 2D structures of molecules generated by different models. Clicking on a row shows the corresponding molecular binding pose in the left viewport. The ligand checkbox in the left side of the viewport can be checked to visualize the original ligand. Besides, users can select the ‘Download all’ or ‘Download selected’ buttons above the table to download all molecules or only the chosen ones, respectively.

#### Overview page

The overview page gives detailed information about the number of molecules generated by each method. Furthermore, it displays the distributions of diverse molecular properties, including Vina score, QED, SA and so on. Additionally, we conducted statistical analysis of bond angles and dihedral angles and subsequently plotted their kernel density estimation diagrams on this page. Besides, we have provided the download links for all trained models and the open-source codes at the end of this page. Moreover, a brief suggestion for model selection is given at the end of the page. Please refer to https://cmach.sjtu.edu.cn/drug/target/ for more detailed information.

## Materials and Methods

Protein target-based molecular generation models can be classified into two groups: autoregressive models and non-autoregressive models, following the paradigm of molecular generation. Common examples of autoregressive models are recurrent neural networks, transformers and graph-based models. These models generate molecules sequentially, atom by atom or fragment by fragment. In contrast, non-autoregressive models, such as diffusion models, generate molecules with a predefined number of atoms. Diffusion models are trained to generate valid molecules by gradually denoising them from random noise. The subsequent section will provide a comprehensive overview of these models.

### AlphaDrug

AlphaDrug (8) is a cutting-edge approach to *de novo* drug design that generates high-affinity molecular drug candidates for a given protein in an autoregressive manner. The model excels in representation learning on protein target and ligand information and employs an efficient heuristic search via Monte Carlo tree search (MCTS) to reduce the computational complexity of exploring the vast search space of possible drug molecules. To co-embed protein targets and molecules, an improved transformer network has been designed. In the transformer variant, AlphaDrug employs a hierarchical skip-connection structure from protein encoders to molecular decoders to enhance feature transfer. The transformer variant calculates the probability of the next atom based on the protein target and molecular intermediates, which guides MCTS to model the molecule generation process symbol by symbol. Additionally, MCTS is guided by a value function implemented by the docking program, reducing the likelihood of selecting paths with low docking values.

### SBDD

SBDD (5) consists of a 3D graph-based generative model and an autoregressive sampling algorithm that work together to generate valid and diverse molecules with high binding affinity to specific targets. Given 3D information about a binding site as context, the model estimates the probability distribution of atomic positions in 3D space. To ensure that the distribution is rotationally invariant to the context, SBDD employs rotationally invariant graph neural networks to extract informative representations of the atoms. The autoregressive sampling algorithm generates 3D molecules by starting with a context consisting of only binding sites. At each step, it samples one atom from the distribution and adds it to the context for the next step until there is no space for new atoms.

### Pocket2Mol

Pocket2Mol (6) is an E(3)-equivariant network that utilizes vector-based neurons and geometric vector perceptrons as basic building blocks for learning chemical and geometric constraints imposed by protein pockets. It consists of two parts: (i) a new graph neural network that captures spatial and bonding relationships between bound pocket atoms; (ii) an efficient conditional 3D coordinate sampling algorithm that samples 3D coordinates directly from the learned Gaussian mixed distribution without relying on Markov chain Monte Carlo. First, the model predicts the frontier atoms of the current molecular fragments. Second, it samples an atom from the frontier set as the focal atom. Third, it predicts the relative position of the new atom using the model’s position predictor based on the focal atom. Finally, the model’s atom element predictor and bond type predictor predict the probabilities of the element types and bond types with existing atoms, and a new atom is added to the current molecular fragments until no frontier atom can be found.

### GraphBP

GraphBP (9) is a method used to generate 3D molecules that can bind to a given protein. The process involves placing atoms of a specific type and location one by one in a given binding protein pocket. A 3D graph neural network is utilized at each step to extract intermediate contextual information, which includes the given binding site and the atoms placed in the previous steps. A local reference atom is then selected based on the designed auxiliary classifier, and a local spherical coordinate system is constructed to maintain ideal equivariance properties. Finally, GraphBP generates the atomic type and relative position with respect to the local reference atom to place the new atom. This method can capture the 3D geometry and chemical interactions of protein–ligand complexes, place atoms without discretization of 3D space and maintain equivariance during generation.

### TargetDiff

TargetDiff (11) is a diffusion-based model that generates molecules that can bind to a given target in a non-autoregressive manner. The model employs an E(3)-equivariant network to learn the joint denoising process of continuous atomic coordinates and discrete atomic types by utilizing atomic-scale context. Moreover, the model has global translation and rotation-invariant likelihood with respect to binding complexes. TargetDiff can also serve as an unsupervised feature extractor, allowing for the extraction of representative features for molecules without requiring retraining. These features can provide robust signals for estimating the binding affinity between sample molecules and the target protein of interest.

### DiffSBDD

DiffSBDD (10) is also a diffusion-based model, which utilizes an E(3)-equivariant graph neural network to learn the denoising process. Two schemes are proposed to generate novel molecule conditioning on protein pockets: protein-conditioned generation and ligand-inpainting generation. The former treats proteins as a fixed environment, and then the model learns to denoise valid molecules from Gaussian noise. The latter mimics the joint distribution of protein–ligand complexes without fixing the proteins. Specifically, the authors trained a diffusion model to denoise noisy molecules and noisy proteins simultaneously. To regulate the denoising process for proteins and align it with the target proteins, at each denoising step, the latent representations of the protein pockets are replaced by the predefined noise of the given protein pockets. In this way, molecular generation of a specified protein pocket can be achieved. We adopt the protein-conditioned generation scheme in our database due to the comparable performance between two schemes.

## Discussion

We have established a comprehensive drug database containing 3D protein–ligand pairs, with all molecules in the database being generated by six popular deep learning models.

Our aim is providing easy access to novel leading compounds for researchers in the fields of bioinformatics and pharmacology and offering a comprehensive benchmark for computational scientists to explore and compare the performance of various AI models in generating novel molecules. We hope that our platform can assist researchers in accelerating the molecular design process. In the future, we will keep on updating our database with state-of-the-art deep generative models, broaden the range of protein targets and provide more in-depth analysis on the performance of each model. We believe that the field of AI-designed molecules has great potential and is still in its early stages of development.

## Data Availability

All data are available for free online viewing through the DrugGen website https://cmach.sjtu.edu.cn/drug/.
